# Protein C-Terminal Labeling and Biotinylation Using Synthetic Peptide and Split-Intein

**DOI:** 10.1371/journal.pone.0008381

**Published:** 2009-12-21

**Authors:** Gerrit Volkmann, Xiang-Qin Liu

**Affiliations:** Department of Biochemistry & Molecular Biology, Dalhousie University, Halifax, Nova Scotia, Canada; Baylor College of Medicine, United States of America

## Abstract

**Background:**

Site-specific protein labeling or modification can facilitate the characterization of proteins with respect to their structure, folding, and interaction with other proteins. However, current methods of site-specific protein labeling are few and with limitations, therefore new methods are needed to satisfy the increasing need and sophistications of protein labeling.

**Methodology:**

A method of protein C-terminal labeling was developed using a non-canonical split-intein, through an intein-catalyzed *trans*-splicing reaction between a protein and a small synthetic peptide carrying the desired labeling groups. As demonstrations of this method, three different proteins were efficiently labeled at their C-termini with two different labels (fluorescein and biotin) either in solution or on a solid surface, and a transferrin receptor protein was labeled on the membrane surface of live mammalian cells. Protein biotinylation and immobilization on a streptavidin-coated surface were also achieved in a cell lysate without prior purification of the target protein.

**Conclusions:**

We have produced a method of site-specific labeling or modification at the C-termini of recombinant proteins. This method compares favorably with previous protein labeling methods and has several unique advantages. It is expected to have many potential applications in protein engineering and research, which include fluorescent labeling for monitoring protein folding, location, and trafficking in cells, and biotinylation for protein immobilization on streptavidin-coated surfaces including protein microchips. The types of chemical labeling may be limited only by the ability of chemical synthesis to produce the small C-intein peptide containing the desired chemical groups.

## Introduction

Proteomics has become one of the fastest growing fields in life science research, and the demand for tools to analyze proteins has increased substantially. Engineering and modifying proteins *in vivo* or *in vitro* can aid the understanding of the biological function and structure-function relationship of proteins. Labeling a protein with a chemical entity, such as a fluorescent group, can facilitate protein characterization with respect to the protein's three-dimensional structure, folding behavior, and interaction with other proteins. Although protein labeling and modification are invaluable tools of protein engineering and extremely useful throughout life science, a major challenge is the addition of a chemical label or moiety site-specifically to a protein. Standard chemical labeling methods often produced mixed populations of labeled proteins, because the targeted amino acid side chains (thiols, carboxyls, amines) were present more than once in the protein of interest. One chemical method can target the N-terminus of a protein specifically [1], however it requires the N-terminus to be solvent-exposed and also depends on the nature of the N-terminal amino acid side chain.

Alternative methods have been developed to label recombinant proteins, with limited successes. In one type of method, certain ligand-binding polypeptide tags were fused with target proteins in recombinant fusion proteins to achieve non-covalent binding of a fluorescent ligand [2], a labeled polypeptide ligand [3], or other small molecule ligands [4]–[6]. These methods are relatively simple to use, but the non-covalent labeling can be unstable, and the presence of the polypeptide tag in the labeled protein may interfere with protein function. In a second type of method, certain polypeptide or protein tags were appended to a target protein, and an enzymatic reaction was used to covalently attach a fluorescent substrate molecule to the polypeptide or protein tag. Enzymes used in these methods included *O^6^*-alkylguanine DNA alkyl transferase [7], phosphopantetheine transferases [8], [9], biotin ligase [10], transglutaminase [11], and sortase [12]. These methods produce stable covalent labeling of proteins, however the polypeptide or protein tags are retained in the labeled protein, which may interfere with protein function. Another elegant method was to incorporate an unnatural or labeled amino acid into a protein during translation [13], which could label or modify a protein seamlessly without an extra polypeptide tag in the labeled protein. However, a specially engineered translation or tRNA charging system is needed for each unnatural amino acid, which increases the technical complexity of this method and limits the type of labels or modifications that can be introduced into proteins.

An intein-based protein cleavage reaction was used cleverly to generate a thioester at the C-terminus of recombinant proteins, which led to specific labeling of the protein C-terminus in a subsequent chemical reaction. Inteins are protein sequences embedded within a host protein, from which the intein catalyzes a protein splicing reaction to excise itself and concomitantly ligate the flanking host protein sequences (N- and C-exteins) [14]. In the absence of a C-extein or splicing, an intein can catalyze a cleavage reaction at its N-terminus, which can be harnessed to create a thioester at the C-terminus of N-extein [15], [16]. This thioester, in the expressed protein ligation method [17], can react with the N-terminal cysteine residue of a polypeptide to achieve protein ligation [17], or with labeled cysteine analogues [18], [19] and bifunctional azides in combination with labeled alkynyl compounds [20] to achieve C-terminal labeling of the N-extein (target protein). A limitation of this method is that it generally requires or introduces a cysteine residue into the labeled protein. In addition, this thioester reaction is not an enzymatic reaction and therefore has some of the limitations common to chemical reactions, such as requiring a relatively high protein concentration.

Protein *trans-*splicing, which is catalyzed by split-inteins, provides another avenue for protein labeling. A split-intein consists of an N-intein and a C-intein, which can associate non-covalently to reconstitute the active intein [21], [22]. A *trans*-splicing reaction takes place between two polypeptides, with one polypeptide consisting of an N-extein fused to the N-intein, and with another polypeptide consisting of the C-intein fused to a C-extein. The *trans*-splicing reaction excises the two inteins and concomitantly ligates the two exteins with a peptide bond. To achieve protein C-terminal labeling, a target protein containing an N-intein has been successfully *trans-*spliced with a synthetic peptide containing a C-intein [23], [24], using the naturally occurring *Ssp* DnaE split-intein. However, the N-intein and C-intein of this and other conventional split-inteins are typically larger than 100 and 35 aa, respectively, which makes it difficult and expensive to produce the synthetic labeling peptide containing the N- or C-intein. Recently a non-canonical split-intein (*Ssp* DnaB S1) was engineered to have a small (11 aa) N-intein and a large (>120 aa) C-intein [25], and it has been used successfully in a *trans*-splicing reaction to label the N-terminus of recombinant proteins *in vitro* [26] and on the surface of mammalian cells [27]. However this *trans*-splicing method cannot be used to label the C-terminus of proteins, because the C-intein is too large to be included in a synthetic labeling peptide.

In this report we aim to produce a method for the C-terminal labeling of recombinant proteins, using an intein-catalyzed *trans*-splicing reaction between a protein and a small synthetic peptide carrying the desired labeling groups. For this purpose, we took advantage of the recently reported non-canonical split-intein (*Ssp* GyrB S11) that consisted of an extremely small (6 aa) C-intein and a large (150 aa) N-intein [28]. We showed for the first time that this S11 split-intein could *trans*-splice proteins with synthetic peptides containing different chemical modifications, although it had been shown before to *trans*-splice a protein with only an unmodified peptide. We achieved site-specific labeling of different recombinant proteins at the C-terminus both *in vitro* and *in vivo* on cell surfaces. Our findings produced a general method for site-specific and efficient chemical labeling or modification of recombinant proteins, which has a number of unique advantages over previously reported methods. Potential usefulness of this method in protein engineering and research are discussed.

## Results

### Protein C-terminal labeling through trans-splicing with a labeled synthetic peptide

**Figure 1:**
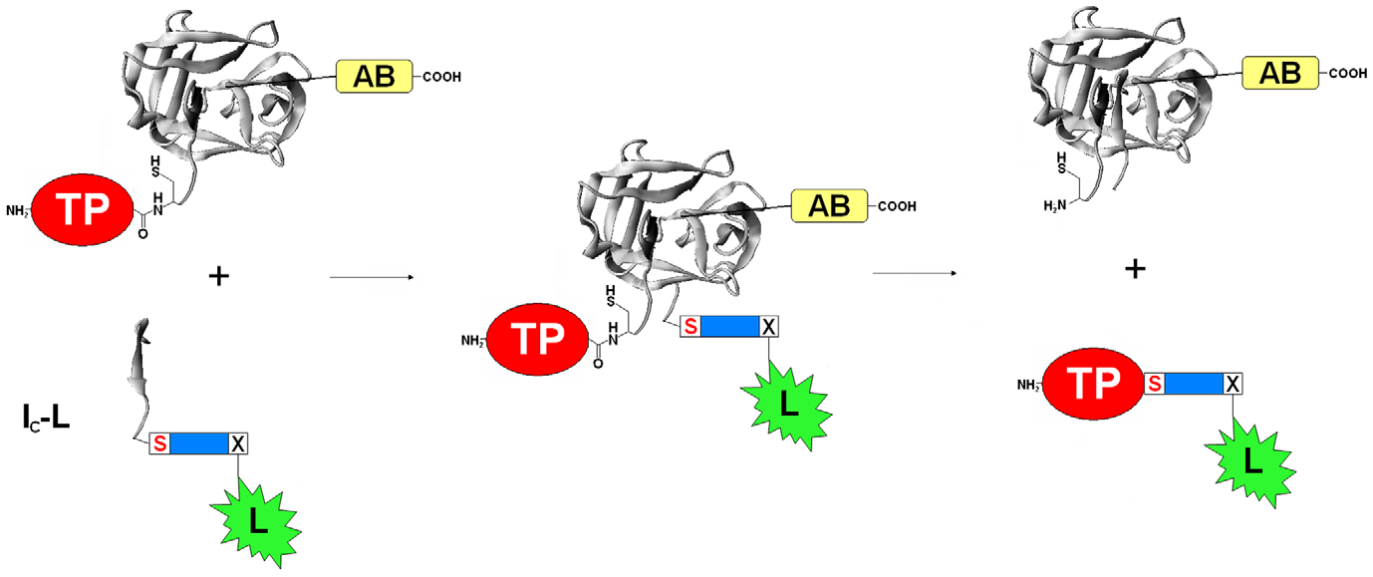
Schematic illustration of the method of protein C-terminal labeling. The precursor protein is a recombinant fusion protein consisting of the target protein (TP) to be labeled, the 150-aa N-intein (I_N_) of the *Ssp* GyrB S11 split-intein for *trans-*splicing, and an affinity binder (AB) for easy purification of the precursor protein. The synthetic peptide consists of the 6-aa C-intein (I_C_) of the *Ssp* GyrB S11 split-intein for *trans-*splicing, followed by a serine residue (highlighted in red), a small linker sequence (blue box), and the labeling group (L) linked to the side chain of a suitable amino acid X. The N-intein and the C-intein are presented in presumed ribbon structures derived from the conserved intein crystal structure of the *Ssp* DnaB mini-intein intein [41], illustrating their association through structural complementation to reconstitute an active intein that catalyzes a *trans-*splicing reaction to produce the labeled protein and the excised inteins.

A strategy of protein C-terminal labeling was designed and shown in Figure 1, by taking advantage of the recently engineered *Ssp* GyrB S11 split-intein [28]. A recombinant precursor protein contains the 150-aa N-intein (I_N_) sandwiched between a target protein and an affinity binder that facilitates purification of the precursor protein. A synthetic peptide contains the 6-aa C-intein (I_C_) followed by a small linker and the labeling group. The short linker begins with a nucleophilic amino acid residue (Serine in this case) required for the splicing reaction. When the precursor protein and the synthetic peptide are incubated together, the large N-intein and the small C-intein are expected to recognize one another, associate non-covalently, form an active intein conformation, and catalyze a protein *trans-*splicing reaction. As a result, the C-terminus of the target protein will be joined through a peptide bond to the small linker followed by the labeling group, with the N-intein (followed by the affinity binder) and the C-intein excised.

**Figure 2:**
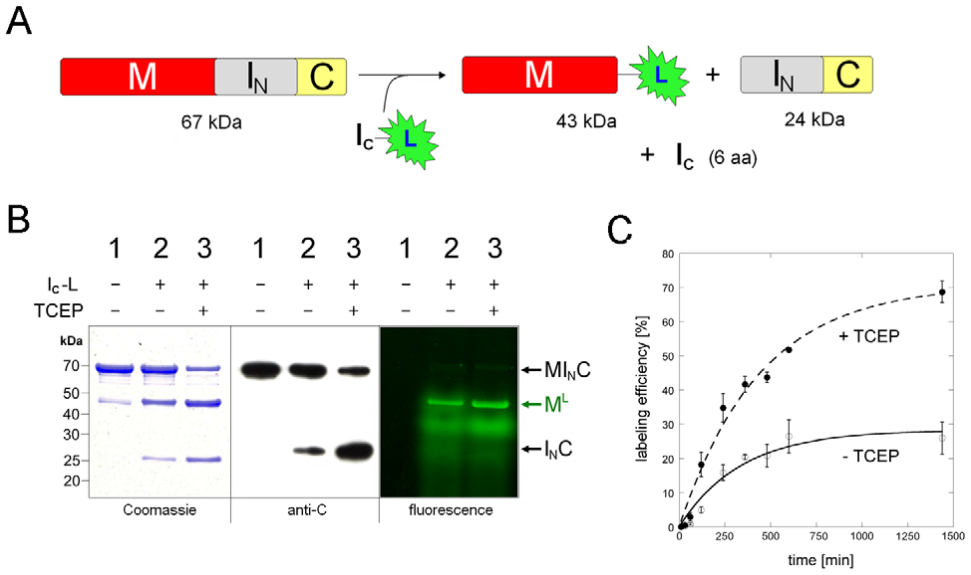
Protein C-terminal labeling with a fluorophore. *A*. Schematic illustration of the labeling reaction. I_N_ and I_C_: components of the *Ssp* GyrB S11 split-intein. M: maltose binding protein as the target protein to be labeled. C: chitin-binding domain as the affinity binder for purification of the precursor protein. L: 5-carboxyfluorescein as the labeling group. In the synthetic peptide I_C_-L, I_C_ is connected to L by the sequence SAGSGK, with L attached to the side chain of the K (lysine) residue. *B*. Analysis of the labeling results. The purified precursor protein was incubated at room temperature for 16 hours, with or without the peptide and the reducing agent TCEP, as indicated. The reaction products were resolved through SDS-PAGE and visualized either by Coomassie staining, by Western blotting using anti-C antibodies, or by fluorescence scan (excitation at 488 nm, filter for 520 nm). Positions of the precursor protein (MI_N_C), the labeled protein (M^L^), and the excised N-intein (I_N_C) are indicated. In lane 1, a minor protein band at the same position as M^L^ is the endogenous *E. coli* maltose binding protein that is known to be co-purified in the amylose affinity chromatography. *C.* Time-course of the reaction between MI_N_C precursor protein and I_C_-L peptide in the presence or absence of TCEP. Labeling efficiency was calculated from densitometry analysis on anti-C Western blots. Error bars represent standard deviations from triplicate experiments.

To demonstrate the above strategy of C-terminal labeling, a precursor protein and a labeling peptide were produced. The precursor protein MI_N_C, which was produced through recombinant DNA and gene expression in *E. coli*, contained a maltose binding protein (M) as the target protein, a chitin binding domain (C) as the affinity binder, and the N-intein sequence (I_N_) in the middle (Figure 2A). The precursor protein was purified using amylose resin, although it could also be purified using chitin beads (see below). The labeling peptide I_C_-L was a 12-aa synthetic peptide having the sequence GVFVHNSAGSGK-**L**, in which GVFVHN was the C-intein, SAGSGK was the C-extein (or linker), and **L** was the labeling group (5-carboxy-fluorescein; λ_exc_ = 492 nm, λ_em_ = 517 nm) linked to the side chain of the lysine (K) residue of the C-extein.

The MI_N_C precursor protein was incubated with the I_C_-L labeling peptide in a splicing buffer at room temperature for 16 hours to achieve *trans-*splicing. Analysis of the reaction products, by SDS-polyacrylamide gel electrophoresis and Western blotting using antibodies against the chitin binding domain (anti-C antibodies), revealed two new protein bands that corresponded in size to the predicted products of the *trans-*splicing reaction (Figure 2B). They were the spliced protein M^L^ (the target protein with the C-terminal labeling group) and the excised N-intein (the I_N_C fragment), while the excised C-intein was too small (6 aa) to be seen on the gel. The M^L^ protein showed the expected green fluorescence in a fluorescence scan (Figure 2B), while the precursor protein and the excised N-intein did not, all as predicted. Diffusive background fluorescence was also visible in the fluorescence scan, but it did not correspond to a labeled protein species, as the Coomassie-stained gel did not show a protein band at the corresponding position.

A significant amount of the precursor protein remained, indicating that the *trans-*splicing reaction did not go to completion. The splicing efficiency, which was defined as the percentage of the precursor protein that underwent splicing, was estimated to be ∼30%, but increased to ∼70% when the reducing agent TCEP was present in the splicing buffer. Kinetic analysis of the labeling reaction between MI_N_C protein and I_C_-L peptide revealed pseudo first-order rate constants *k*_obs_ of (5.0±0.7)×10^−5^ s^−1^ and (3.8±0.6)×10^−5^ s^−1^ in the absence and presence of TCEP, respectively (Figure 2C).

**Figure 3:**
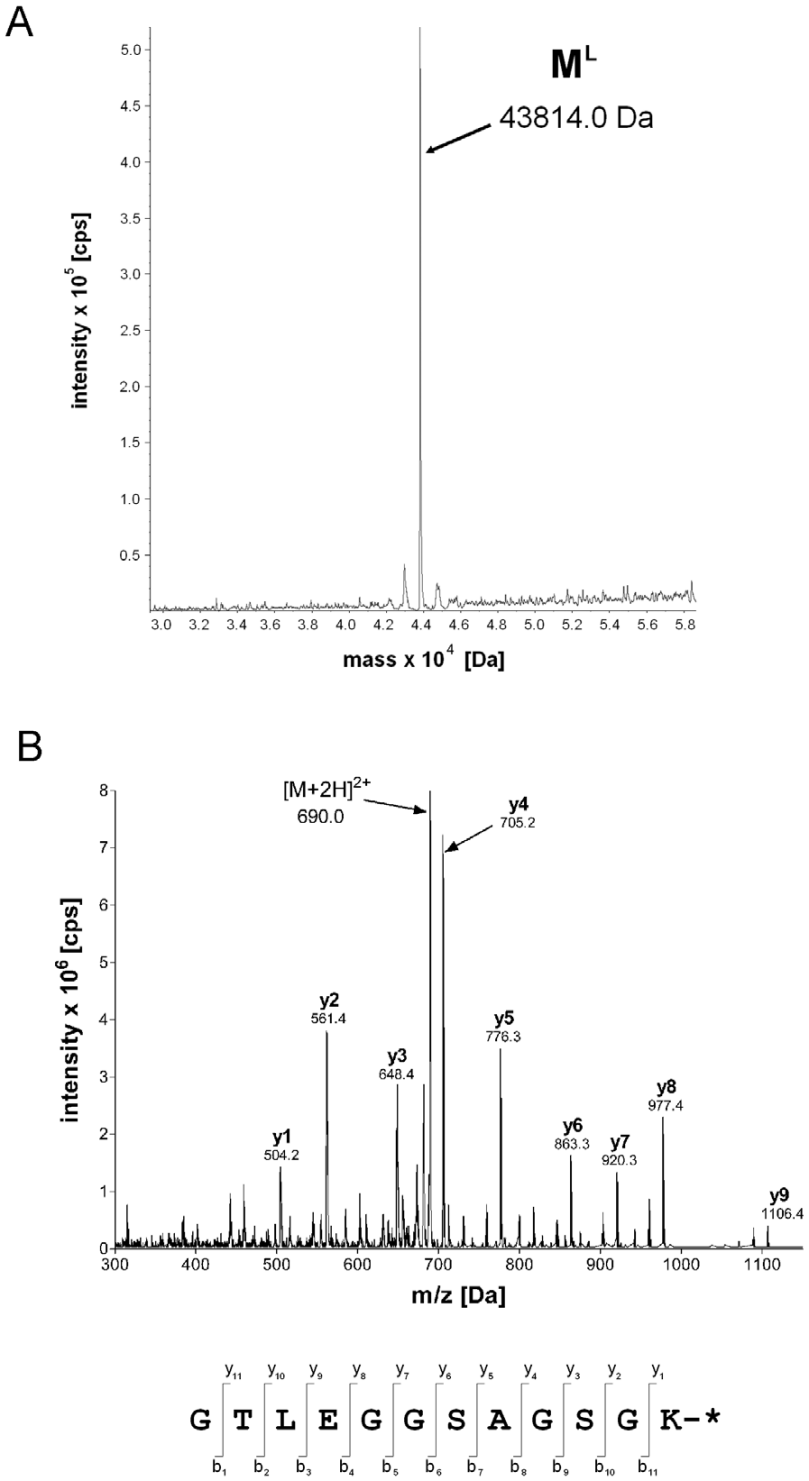
Mass spectrometry identification of the fluorescently labeled maltose binding protein M^L^. *A*. ESI-MS analysis of the labeled protein. The determined molecular weight of 43814 Da was in close agreement with the calculated mass (43830.7 Da) of the labeled protein M^L^. *B*. MS/MS analysis of the labeled protein after trypsin digestion. The deconvoluted spectrum of a 1378-Da peptide corresponded well to the calculated mass (1378.964 Da) of the C-terminal peptide of M^L^. The y-fragment ions of this peptide yielded to the amino acid sequence given below the spectrum, which unambiguously identified the *trans*-spliced peptide at the C-terminus of the labeled M^L^ protein.

The spliced protein M^L^ was further identified through mass spectrometry analyses. An electron spray ionization mass spectrometry (ESI-MS) analysis of the M^L^ protein determined a molecular mass of 43814.0 Da (Figure 3A), which was in close agreement with the calculated mass of 43830.7 Da. After trypsin digestion of the M^L^ protein, an ESI-MS analysis revealed an [M+2H]^2+^ peptide ion having a deconvoluted mass of 1378.0 Da, which corresponded well with the calculated mass (1378.964 Da) of the expected C-terminal trypsin peptide of the spliced protein M^L^. The amino acid sequence of this C-terminal trypsin peptide was determined by tandem mass spectrometry (MS/MS) analysis to be GTLEGGSAGSG (Figure 3B), in which GTLEGG matched the C-terminal sequence of the M protein, and SAGSG matched the C-extein sequence of the I_C_-L peptide, all as expected. The C-terminal Lys residue with the fluorophore was also revealed as a fragment ion of 504.2 Da, which matched closely with the calculated mass of 505.91 Da. All together, these mass spectrometry analyses clearly identified the spliced protein, which confirmed a successful production of the fluorescently labeled M^L^ protein by *trans*-splicing.

### Distinguishing trans-splicing from N-cleavage

Protein *trans-*splicing may sometimes be accompanied by N- or C-cleavages as undesirable side reactions, in which the peptide bond at the intein's N- or C-terminus is broken without forming a new peptide bond as in splicing. In this study, the spliced (labeled) protein band (M^L^) in Figure 2B could possibly be a mixture of the splicing product and the N-cleavage product, because these two protein products had similar molecular masses (43830.72 Da and 42984.47 Da, respectively). To test this possibility, the following experiments were carried out.

**Figure 4:**
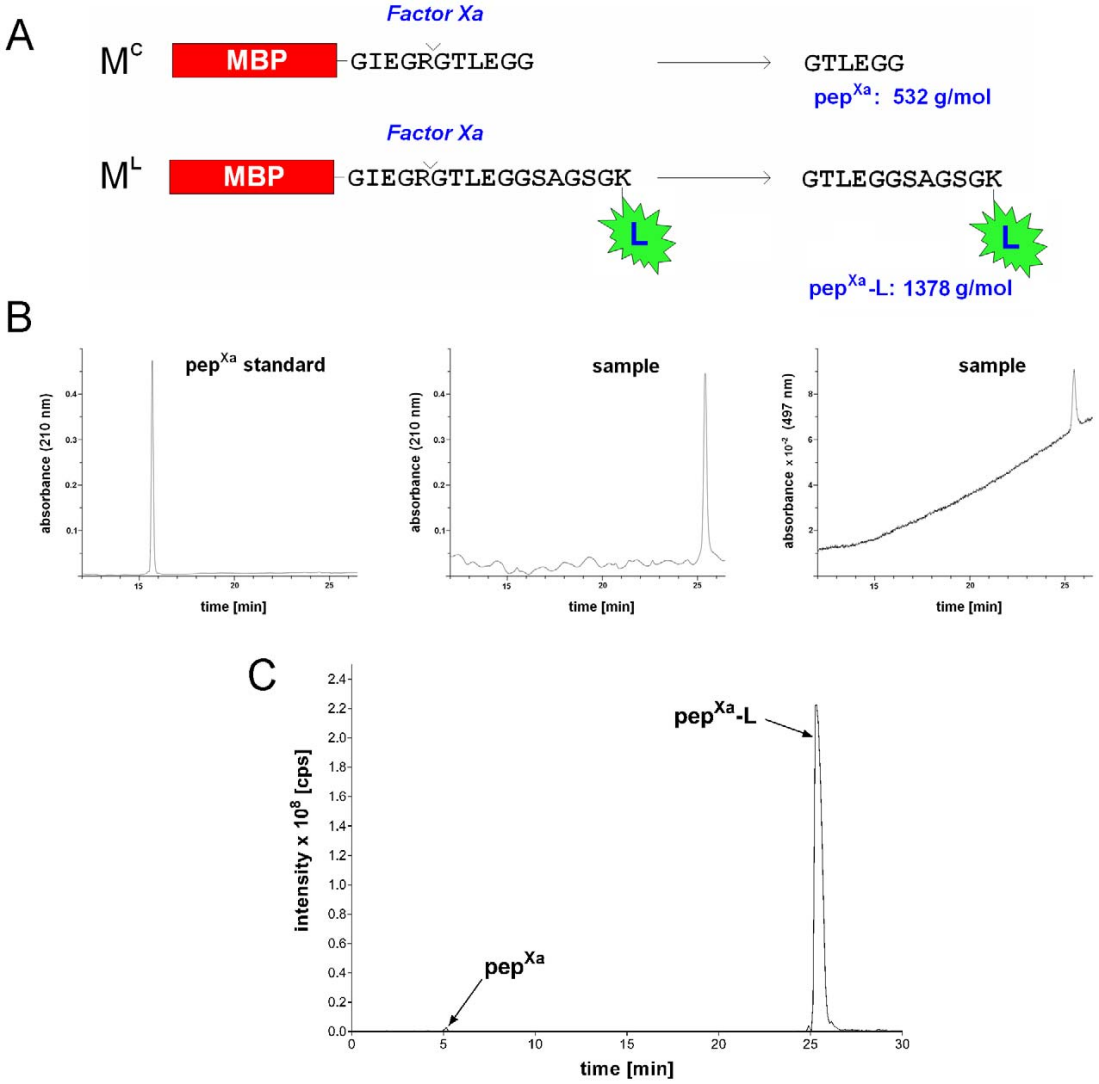
Detection of possible N-cleavage as a side reaction. *A.* Illustration of the detection strategy. The predicted N-cleavage product M^C^ is compared with the *trans-*splicing (C-terminally labeled) product M^L^. Site-specific cleavage of M^C^ and M^L^ with protease Factor Xa releases the C-terminal peptide pep^Xa^ and pep^Xa^-L, respectively. *B*. RP-HPLC analysis. The *left* chromatogram shows a synthetic pep^Xa^ used as a standard. The middle and right chromatograms show the sample peptide detected at 210 nm (*middle*) and 497 nm (*right*). The increasing baseline absorbance at 497 nm over time was most likely due to the absorbance of the increasing concentration of the mobile phase B solution. *C*. MS-coupled HPLC analysis. The pep^Xa^-L was much more abundant than pep^Xa^, and both peptides were identified by their corresponding molecular weights in mass spectrometry analysis (data not shown).

Protein products of the C-terminal labeling reaction were digested with the site-specific protease Factor Xa to produce a short C-terminal peptide. As illustrated in Figure 4A, this peptide from the splicing (C-terminally labeled) product would have a molecular mass of 1378 Da, whereas the peptide from the N-cleavage product would have a molecular mass of 532 Da. This size difference and the highly hydrophobic nature of the fluorescein on the C-terminally labeled peptide allowed easy separation of the two peptides by reverse phase high-performance liquid chromatography (RP-HPLC). As shown in Figure 4B, the RP-HPLC analysis revealed a single strong signal (peak) of the C-terminal labeled peptide corresponding to the splicing product. No signal was observed for the unlabeled peptide corresponding to the N-cleavage product, whose position was predicted using a control peptide, indicating that there was no detectable amount of the N-cleavage product.

Further detection of possible N-cleavage product was also carried out, using a more sensitive method. Protein products of the above C-terminal labeling reaction, after digestion with protease Factor Xa, were analyzed by liquid chromatography (under conditions different from the above RP-HPLC) followed by tandem mass spectrometry (LC-MS/MS). As shown in Figure 4C, the liquid chromatography revealed a large peak corresponding to the labeled peptide from splicing and a tiny peak corresponding to the unlabeled peptide from N-cleavage, with both peptides being identified through MS/MS analysis (data not shown). The relative peak areas of the two peptides were measured to be 7.14×10^7^ and 5.75×10^9^, respectively, which were used to estimate the relative amounts of the spliced (labeled) product and the N-cleavage product. Based on this estimation, the labeling (*trans-*splicing) reaction made up ∼98.7% of the total, while the N-cleavage reaction made up the remaining ∼1.3%.

### Purification of C-terminally labeled protein

**Figure 5:**
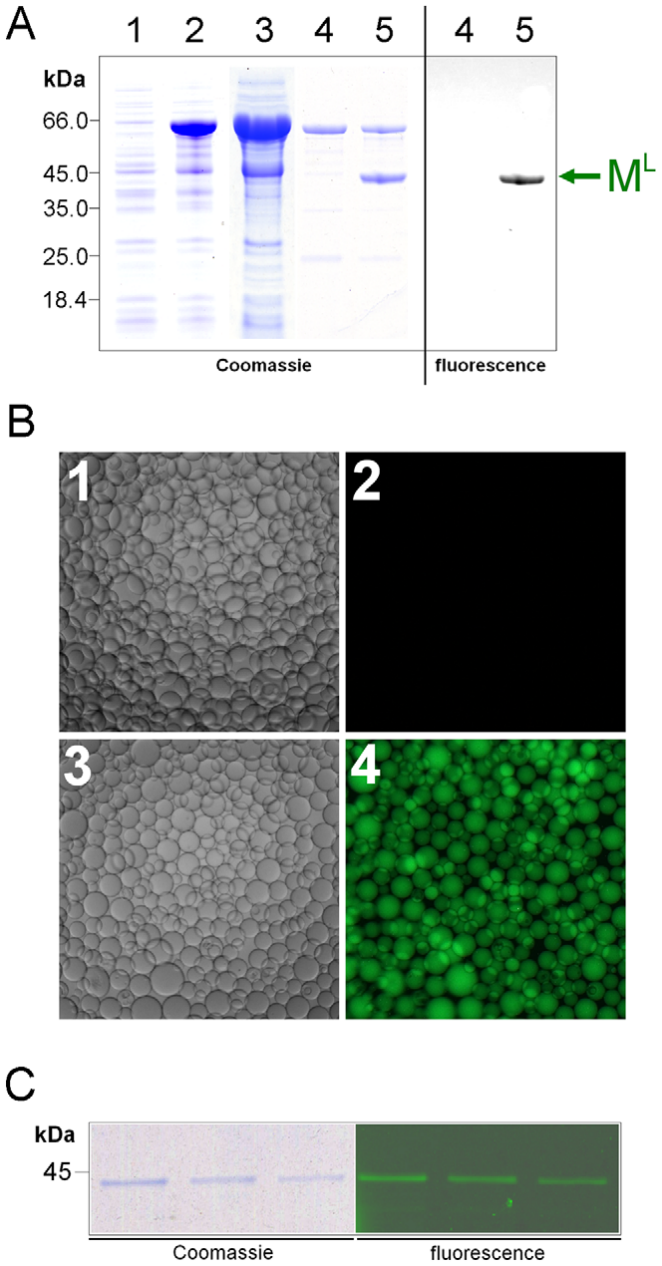
On-column production of M^L^ protein. *A.* The co-purification labeling process was monitored through SDS-PAGE analysis with Coomassie blue staining or fluorescence scanning as indicated. Lanes 1 and 2: total *E. coli* proteins before and after IPTG-induced expression of the precursor protein MI_N_C, respectively. Lane 3: soluble fraction of the cell lysate of lane 2. Lane 4: proteins of lane 3 bound to chitin beads. Lane 5: proteins released from the chitin beads of lane 4 after an overnight incubation with the labeling peptide I_C_-L in the presence of TCEP. *B.* Amylose resin was incubated with (panels 1 and 2) or without (panels 3 and 4) the purified M^L^ protein. Panels 1 and 3 are differential interference contrast images, while panels 2 and 4 are fluorescence images. *C.* SDS-PAGE analysis of the M^L^ protein fractions eluted from the amylose resin of panels 3 and 4 of *B* using a maltose-containing elution buffer.

We then showed that the target protein M could be labeled in and purified from a cell lysate in a single chromatographic step, which takes advantage of the affinity binder (chitin-binding domain in this case) fused to the C-terminal of the N-intein in the MI_N_C protein. To achieve this, the MI_N_C protein in an *E. coli* cell lysate was immobilized on chitin beads, the unbound proteins were washed away, and the beads were subsequently incubated with the labeling peptide I_C_-L. The subsequent *trans*-splicing reaction produced and simultaneously released the labeled protein M^L^ from the chitin beads (Figure 5A). The elution fraction also contained some MI_N_C precursor protein, which likely fell off the chitin resin during the prolonged labeling incubation and could be removed by a subsequent passage of the sample through fresh chitin resin. The resulting purified M^L^ protein was able to bind to amylose resin (Figure 5B) and could subsequently be eluted with maltose-containing buffer (Figure 5C), which showed that the covalent attachment of 5-carboxyfluorescein to the C-terminus of the maltose binding protein did not interfere with the protein's biochemical function.

### Protein C-terminal biotinylation through peptide splicing

Because protein biotinylation can be an effective way of protein immobilization on streptavidin-coated protein chips [19], [30], we tested whether our labeling method could achieve C-terminal biotinylation of recombinant proteins. For this purpose, we produced a synthetic peptide I_C_-B having the sequence GVFVHNSAGSK-(Biotin), in which GVFVHN was the C-intein, SAGSK was the C-extein (or linker), and biotin was linked to the side-chain of the C-terminal Lys residue. Two new precursor proteins were also constructed, in addition to the MI_N_C precursor protein described above, in order to assess the versatility of the C-terminal biotinylation reaction. The EI_N_H precursor protein contained an enhanced Green Fluorescent Protein (E) as the target protein, whereas in GST-ACP-I_N_C the target protein was a fusion of a Glutathione-S-transferase (GST) and an acyl carrier protein (ACP). To facilitate protein purification and identification, the *Ssp* GyrB S11 I_N_ sequence was followed either with a hexa-histidine tag (H) in the EI_N_H protein or a chitin binding domain (C) in the GST-ACP-I_N_C protein.

**Figure 6:**
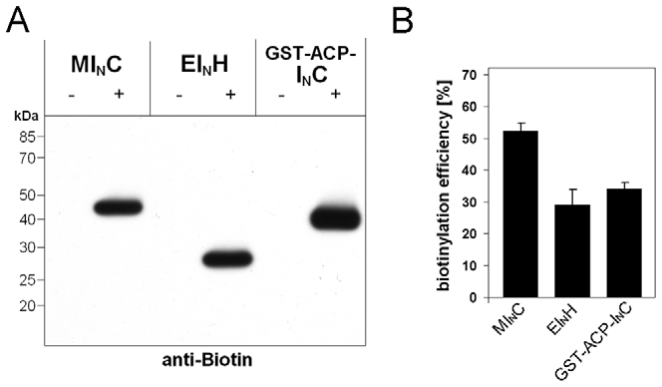
C-terminal biotinylation using the *Ssp* GyrB S11 split-intein. *A.* The three precursor proteins (MI_N_C, EI_N_H, and GST-ACP-I_N_C) were incubated with (+) or without (−) the peptide I_C_-B in the presence of 0.1 mM TCEP for 18 h at room temperature, and the reaction products were analyzed by Western blotting using antibodies against biotin. From the three precursor proteins, the three target proteins for biotinylation were a maltose binding protein (M, 43 kDa), an enhanced green fluorecent protein (E, 27 kDa), and a glutathione-S-transferase/acyl carrier fusion protein (GST-ACP, 39 kDa), respectively. *B.* Efficiency of C-terminal biotinylation of the three target proteins as determined by densitometry analysis on Western blots using anti-C antibodies (for MI_N_C and GST-ACP-I_N_C) and anti-H antibodies (for EI_N_H). Error bars represent standard deviations from triplicate experiments.

A *trans*-splicing reaction of each precursor protein with the I_C_-B peptide produced a biotinylated protein, as revealed by Western blot analysis using antibodies against biotin (Figure 6A). The apparent sizes of these three biotinylated proteins matched closely with their predicted molecular weights, which were 43 kDa, 27 kDa, and 39 kDa, respectively, for target proteins generated from the MI_N_C, EI_N_H, and GST-ACP-I_N_C precursor proteins. To estimate the efficiencies of the protein biotinylation, Western blots were carried out using antibodies against the chitin binding domain (C) for reactions involving MI_N_C and GST-ACP-I_N_C, or using antibodies against the hexa-histidine tag (H) for reactions involving EI_N_H. After estimating the quantities of the precursor protein and the biotinylated protein on these Western blots, the efficiencies of the protein biotinylation was calculated to be 30–50% for the three different precursor proteins (Figure 6B).

**Figure 7:**
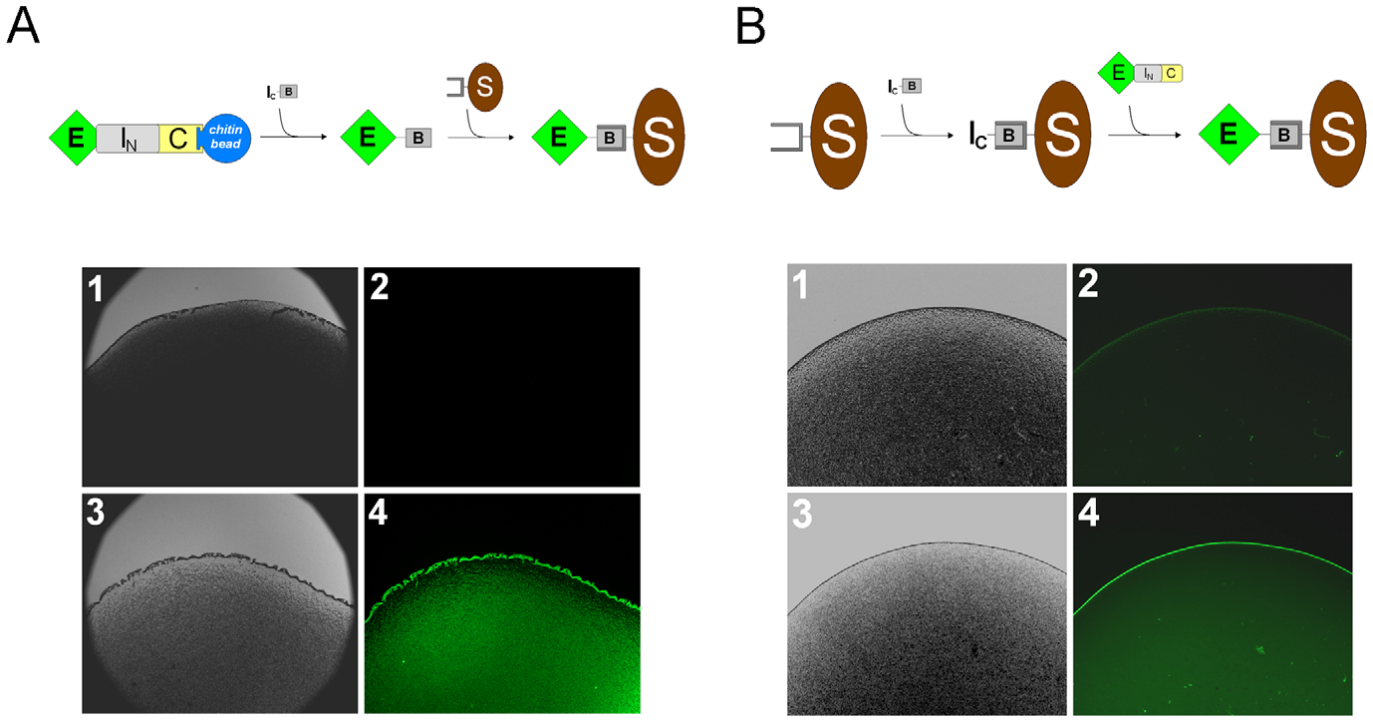
Immobilization of biotinylated enhanced green fluorescent protein (E^B^) to a solid surface. *A,* The biotinylation-purification method. The precursor protein (EI_N_C) in a cell lysate is first bound to chitin beads through its chitin binding domain (C). After washing away the unbound proteins, the beads are incubated with the peptide I_C_-B that consists of I_C_ followed by the sequence SAGSK with biotin linked to the lysine (K) side chain. A *trans-*splicing reaction produces the biotinylated target protein (E^B^) that can bind to streptavidin-coated beads (S). In the experimental proof, streptavidin-coated beads were incubated either with the EI_N_C precursor in a cell lysate (panels 1 and 2) or with the biotinylated and purified E^B^ protein (panels 3 and 4). Panels 1 and 3 are differential interference contrast images, while panels 2 and 4 are fluorescence micrographs (45 ms exposure time). *B,* The biotinylation-fixation method. The I_C_-B peptide is first bound to streptavidin-coated beads and then incubated with the precursor protein EI_N_C in a cell lysate to achieve *trans-*splicing, and the resulting biotinylated target protein (E^B^) is automatically fixed to the beads. In the experimental demonstration, streptavidin-coated beads with (panels 3 and 4) or without (panels 1 and 2) the pre-bound I_C_-B peptide were incubated with the EI_N_C protein in an *E. coli* cell lysate for *trans*-splicing. After washing away unbound proteins, the beads were photographed as differential interference contrast images (panels 1 and 3) or as fluorescence images (panels 2 and 4, 600 ms exposure time).

To test whether the C-terminally biotinylated protein could be immobilized on a streptavidin-coated surface, we carried out co-purificational C-terminal biotinylation of the enhanced green fluorescent protein (Figure 7A). The precursor protein EI_N_C was first bound to chitin beads (column) through its chitin binding domain and then incubated with the I_C_-B peptide to allow on-column *trans*-splicing, which biotinylated and simultaneously released the target protein. When this biotinylated protein was incubated with streptavidin-coated beads, strong green fluorescence was observed on the surface of the beads, indicating binding of the protein to the beads. In a negative control, no green fluorescence appeared on the bead surface after incubation with *E. coli* cell lysate containing the EI_N_C precursor protein without biotinylation.

We then tested whether the protein C-terminal biotinylation and the immobilization on a solid surface could be achieved in one step when the I_C_-B peptide was pre-bound to the streptavidin-coated surface (Figure 7B). Streptavidin-coated beads were first incubated with the I_C_-B peptide to allow binding. After washing away any excessive unbound peptide, the beads were incubated with *E. coli* cell lysate containing the EI_N_C protein to allow *trans*-splicing. After washing away any unbound protein, the beads showed bright green fluorescence, indicating that the enhanced green fluorescent protein was immobilized to the beads as a result of *trans*-splicing between the EI_N_C precursor protein and the pre-bound I_C_-B peptide. In a negative control, no bright green fluorescence was observed when the same cell lysate was incubated with streptavidin-coated beads without the pre-bound I_C_-B peptide, and the dim green fluorescence was due to autofluorescence of streptavidin beads after the prolonged exposure time.

### Fluorescent labeling of a cell surface receptor protein

**Figure 8:**
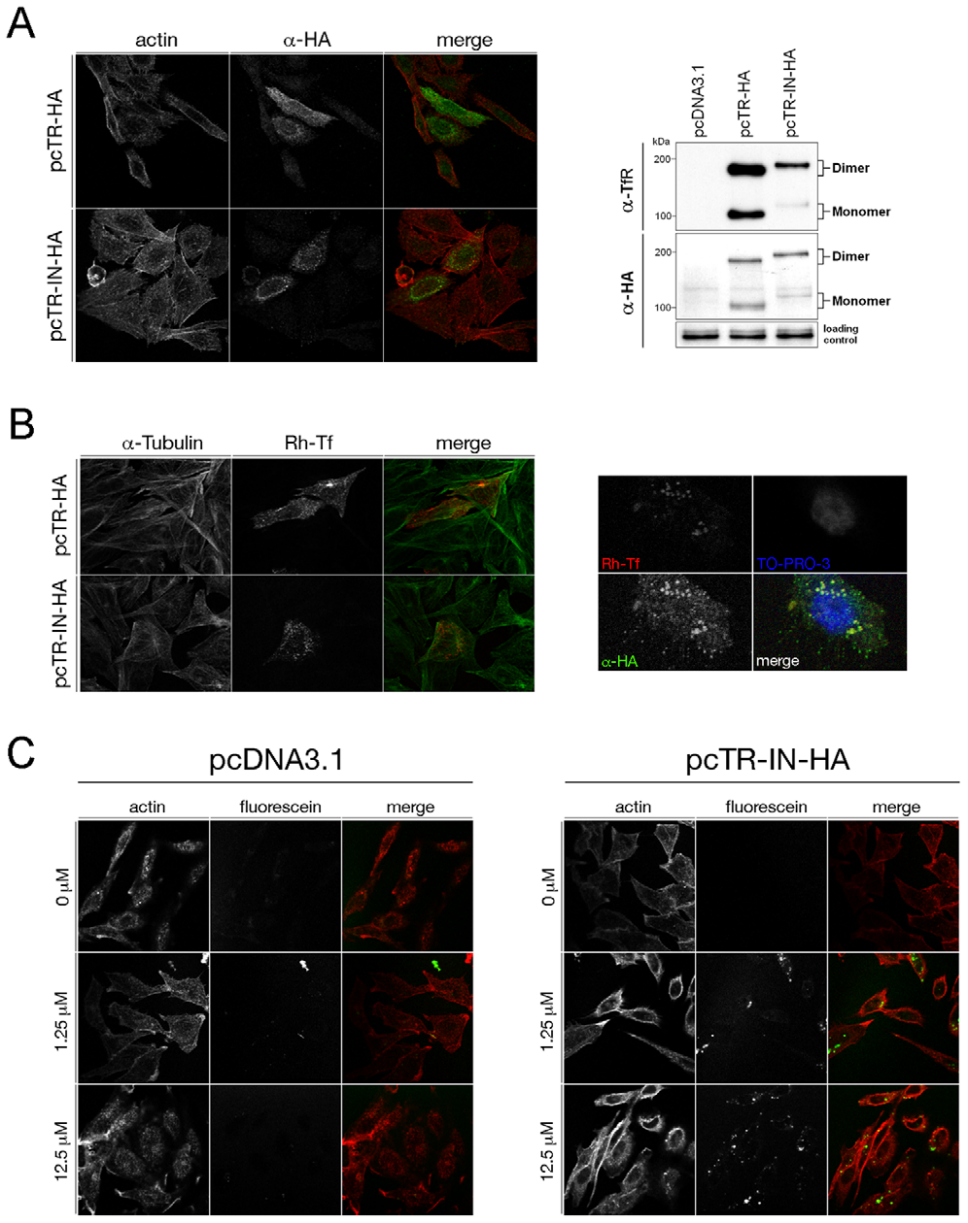
Labeling of transferrin receptor (TfR) protein on live cells. *A.* Expression of the TfR-intein fusion protein. CHO-TRVb cells, which are Chinese hamster ovary cells deficient for endogenous TfR, were transfected with plasmid pcTR-HA expressing a human TfR fused to a C-terminal HA tag, plasmid pcTR-IN-HA expressing the TfR fused to I_N_ and the HA tag (TfR-I_N_-HA fusion protein), or plasmid pcDNA3.1 as an empty vector, as indicated. On the left are confocal microscopy images of immunofluorescence using antibodies against the HA tag (α-HA), and the cells were counterstained with rhodamine-phalloidin for actin. On the right are Western blots using α-HA or α-TfR antibodies, with the monomeric and dimeric forms of the receptor proteins indicated. *B*, Function of the TfR-intein fusion protein. On the left, CHO-TRVb cells transfected with the indicated plasmids were incubated with rhodamine-labeled transferrin (Rh-Tf) to show function of the TfR-intein fusion protein in cellular uptake of transferrin. The cells were counterstained using antibodies against tubulin. On the right, cells transfected with the pcTR-IN-HA plasmid were incubated with Rh-Tf and also visualized by immunofluorescence using α-HA antibodies, which showed co-localization of the TfR-I_N_-HA fusion protein with Rh-Tf. The cells were counterstained with TO-PRO-3 for the nucleus. *C.* Observation of labeling of TfR. CHO-TRVb cells transfected with the indicated plasmids were incubated with increasing amounts (as indicated) of the fluorescent I_C_-L peptide (described in Figure 2). Only cells expressing the TfR-intein fusion protein from the pcTR-IN-HA plasmid showed discrete green fluorescent spots, indicating successful labeling of TfR. The cells were counterstained with rhodamine-phalloidin for actin in the confocal microscopy.

We tested whether the C-terminal labeling method could label proteins on the surface of live cells under nearly physiological conditions. Human transferrin receptor (TfR) was chosen as the target protein, because the C-terminus of this well characterized protein is on the outer surface of the cell membrane. A recombinant fusion protein (TfR-I_N_-HA), which consisted of TfR followed by the *Ssp* GyrB S11 I_N_ sequence and a C-terminal HA epitope, was successfully produced in TfR-deficient Chinese hamster ovary cells (CHO-TRVb [31]) through plasmid transformation, as evidenced by immunofluorescence and Western blotting (Figure 8A). Presence of this TfR-I_N_-HA protein on the cell surface, which was a prerequisite for the C-terminal labeling reaction, was further confirmed by the cellular uptake of rhodamine-labeled transferrin Rh-Tf (Figure 8B).

To carry out fluorescent labeling of the transferrin receptor, the above CHO-TRVb cells expressing the TfR-I_N_-HA fusion protein were incubated with an increasing amount of I_C_-L peptide. After washing away any excessive I_C_-L peptide, confocal microscopy revealed discrete green fluorescent spots associated with the cells (Figure 8C), indicating a successful fluorescent labeling of the transferrin receptor protein through a *trans*-splicing reaction between the TfR-I_N_-HA fusion protein and the I_C_-L peptide. The green fluorescence was sometimes observed inside the cell additional to the cell surface, and this was consistent with endocytic internalization of the labeled transferrin receptor protein, which is a known function of the transferrin receptor protein [32], [33]. In a negative control, cells transfected with empty vector (pcDNA3.1), did not show any green fluorescence.

## Discussion

We have demonstrated an efficient method of protein C-terminal labeling, by *trans-*splicing recombinant proteins with synthetic peptides containing the labeling moieties. This *trans*-splicing method is uniquely suitable for protein C-terminal labeling, in contrast to a previously reported *trans*-splicing method using the S1 split-intein for protein N-terminal labeling [26], [29]. This method has the following three advantages, when compared to previous *trans*-splicing methods using conventional split-inteins having a larger (>30 aa) C-intein. First, it uses an extremely small (6 aa) C-intein that can be accommodated in a synthetic peptide more easily and economically, particularly when the synthetic peptide needs to contain large and complex labeling groups. Second, the 6-aa S11 C-intein is free of amino acid residues commonly used for chemical modification (Lys, Cys, Glu), therefore any one or a combination of these residues may be added after the C-intein in the synthetic peptide to serve as specific site(s) for chemical attachment of desired labeling group(s). Third, the N-intein of the S11 split-intein resembles more closely the intact intein and therefore is less likely to misfold in the precursor protein. In a previously reported “cysteine tag” method of intein-mediated protein C-terminal labeling [34], [35], a cysteine-containing recombinant protein was used as the label carrier. In contrast, the *trans-*splicing method of this study uses a synthetic peptide as the label carrier, which can be advantageous in terms of the larger number of labeling moieties that can be chemically synthesized. When compared to the expressed protein ligation (EPL) method [17], the *trans-*splicing method of this study does not require a Cys residue at the site of the protein-peptide ligation, and it is completely an enzymatic reaction that can work with very low protein concentrations under gentle or physiological conditions. Consistent with this notion, the labeled proteins in this study retained their original biochemical functions (ligand binding, epifluorescence), and a receptor protein was successfully labeled on the membrane surface of live mammalian cells.

The C-terminal labeling method of this study adds only a minimal amount of extra sequence to the labeled protein, in contrast to some other labeling methods that add a large polypeptide sequence (up to hundreds of amino acids) to the labeled protein [7]–[10]. This can be a considerable advantage, because adding a minimal extra sequence minimizes the risk of hindering normal functions of the labeled protein. In the *trans*-splicing method of this study, only the small C-extein is *trans-*spliced onto the C-terminus of the target protein, while the C-intein and the N-intein are excised and not present in the labeled protein. In the I_C_-L peptide used in this study, the 6-aa C-extein sequence was SAGSGK, in which the first S residue was required for the *trans-*splicing reaction, and the last K residue was used to attach the labeling group. The middle four residues were included as a linker to provide additional distance between the C-intein and the bulky 5-carboxyfluorescein, but this additional distance might not be necessary when the labeling group is small and does not interfere with the intein function. Indeed, protein C-terminal biotinylation was achieved in this study using the I_C_-B peptide having only a 3-aa linker.

The C-terminal labeling method of this study showed a relatively high (75%) efficiency of the desired *trans*-splicing reaction with only a negligible amount (<2%) of the undesired N-cleavage side reaction, when compared to previous *trans-*splicing methods using other inteins. For example, much lower (∼40%) efficiencies were reported for the *Ssp* DnaE split-intein [36], the *Sce* VMA split-intein [37], and the *Ssp* DnaB S1 split-intein [26], and the latter intein also exhibited a high level (20–40%) of the undesired N-cleavage side reaction [26], [29]. The labeling reaction in this study showed a pseudo first-order rate constant of (3.8±0.6)×10^−5^ s^−1^ under optimal conditions, which is within the range of previously reported rate constants of split-inteins used in protein labeling reactions [26], [34], [38]. We used 45–250 micromolar concentrations of the labeling peptides to achieve high efficiencies of the protein labeling, which is similar to the other protein *trans*-splicing reactions. Decreasing the peptide concentration to the nanomolar range did not produce a detectable amount of protein labeling under the conditions used in this study (data not shown).

Our *trans-*splicing method also exhibited generality and versatility for protein C-terminal labeling. We have successfully demonstrated this method with three different target proteins (MBP, eGFP, and GST-ACP) and two different labeling groups (fluorescein, biotin). We showed that the N-intein could tolerate the C-terminal fusion of an affinity binder for easy purification of the precursor protein. The affinity binder was a chitin-binding domain or a hexa-histidine tag in this study, but potentially it could also be others such as glutathione-S-transferase. The affinity binder is conveniently removed along with the N-intein in the subsequent *trans-*splicing reaction for C-terminal labeling. This allowed us to carry out the labeling reaction with the precursor still bound to a protein purification column, which labeled and simultaneously released the target protein from the column, making this the first method of labeling and purifying a target protein in one step. We demonstrated C-terminal biotinylation of a protein either in solution or on a solid surface, which allowed site-specific (i.e. C-terminal) immobilization of the biotinylated target protein on a streptavidin-coated surface. We also achieved protein-specific biotinylation and immobilization in a complex protein mixture (*E. coli* cell lysate) without purifying the precursor protein, which is a considerable advantage of this method for high-throughput protein immobilization on protein chips. Finally, we were able to label a membrane receptor protein on the surface of a live mammalian cell, because the labeling (*trans*-splicing) reaction could occur under nearly physiological conditions.

In conclusion, we have produced an efficient and useful method for the C-terminal labeling of recombinant proteins, using an intein-catalyzed *trans*-splicing reaction between a protein and a small synthetic peptide carrying the desired labeling groups. This method has a number of unique advantages over previously reported methods and is expected to have many potential applications in protein engineering and research. Adding a fluorophore to the C-terminus of a protein, both in solution and on the membrane surface of a live cell, can be a useful tool for monitoring protein folding, location, and trafficking in cells. The C-terminal biotinylation of a protein can be a superior way of protein fixation on streptavidin-coated surfaces including protein microchips used in proteomics. Potentially many other chemical groups can be added to the C-terminus of proteins using this method, which is limited only by the ability of chemical synthesis to produce the small C-intein peptide containing the desired chemical groups.

## Materials and Methods

### Plasmid construction and protein purification

Plasmid pMI_N_C was constructed by replacing the *Ssp* DnaB intein sequence in the pMST plasmid [39] with coding sequence of the *Ssp* GyrB S11 I_N_, and replacing the thioredoxin sequence with coding sequence of the chitin binding domain. Plasmid pTEI_N_C was constructed by replacing the double-intein ORF of the pTWIN vector (New England Biolabs) with the coding sequences of enhanced green fluorescent protein, followed by the *Ssp* GyrB S11 I_N_ and the chitin binding domain. In plasmid pTEI_N_H, the chitin binding domain coding sequence of pTEI_N_C was replaced with a sequence for a hexahistidine tag. The plasmid pGST-ACP-I_N_C was obtained by cloning the coding sequence for the *Ssp* GyrB S11 I_N_ (followed by the chitin binding domain) downstream of the *V. harveyi* ACP in plasmid pGEX-F50W [40]. Plasmid pcTR-IN-HA was constructed by fusing the *Ssp* GyrB S11 I_N_ with a C-terminal HA epitope tag to the coding sequence of the human transferrin receptor, and cloning the fusion gene into the mammalian expression vector pcDNA3.1. Plasmid pcTR-HA is similar to pcTR-IN-HA but lacks the coding sequence of the *Ssp* GyrB S11 I_N_ fragment.

The MI_N_C precursor protein was expressed from plasmid pMI_N_C in *E. coli* DH5α cells and purified by amylose affinity chromatography according to the manufacturer's guidelines (New England Biolabs). The EI_N_H and GST-ACP-I_N_C proteins were over expressed in BL21(DE3)pLysS cells and purified on Ni-NTA agarose (Qiagen) and Glutathione Sepharose 4B (GE Healthcare), respectively, using standard procedures.

### *In vitro* protein labeling

For fluorescent labeling, ∼5 µM MI_N_C protein was incubated with 125 µM peptide I_C_-L (sequence: GVFVHNSAGSGK-(5-carboxyfluorescein) purchased from New England Peptide) in optimized Splicing Buffer (oSB: 20 mM Tris-HCl, 250 mM NaCl, 1 mM EDTA; pH 8.5) in the presence or absence of 0.1 mM TCEP for 16 h at room temperature. Reactions were stopped with SDS sample buffer, and samples were run on a 12.5% SDS-PAGE gel in the dark. After completion of electrophoresis, the gel was equilibrated in 10% ethanol/7% acetic acid and scanned for fluorescence using the Typhoon 9410 equipment (Amersham Biosciences) with excitation wavelength of 488 nm and an emission filter for 520 nm. Afterwards, the gel was stained with Coomassie Brilliant Blue and destained using standard procedures. For biotinylation, ∼5 µM precursor proteins were incubated with 250 µM peptide I_C_-B (sequence: GVFVHNSAGSK-biotin, purchased from New England Peptide) in oSB in the presence of 0.1 mM TCEP. Reactions were separated by 12.5% SDS-PAGE, and subjected to Western blotting using mouse anti-Biotin antibody (Sigma Aldrich). Additional Western blotting was carried out with antibodies against the chitin binding domain (mouse anti-C antibody) and the His-tag (mouse anti-H antibody), which were purchased from New England Biolabs and Sigma Aldrich, respectively. All primary antibodies were used in combination with a secondary rabbit-anti mouse IgG HRP-linked antibody (GE Healthcare). Chemiluminescence was performed with the ECL Plus Western Detection Kit according to manufacturor's guidelines (GE Healthcare).

### Simultaneous protein purification and labeling

Precursor proteins MI_N_C and EI_N_C were expressed in *E. coli* DH5α and BL21(DE3), respectively, and the cells were lysed in oSB. The soluble fraction of the cell lysate was loaded onto chitin resin. After washing away unbound proteins with oSB, the resin was incubated in oSB containing 0.1 mM TCEP and either 45 µM I_C_-L (for MI_N_C protein) or 125 µM I_C_-B (for EI_N_C protein, sequence: GVFVHNSAGSK-biotin, purchased from GenScript), and the incubation was continued overnight at room temperature. Proteins released from the resin were collected and further purified using a small amount of freshly equilibrated chitin resin. The purified protein was then concentrated using Amicon Ultra Centrifugal Filter Devices (Millipore) and analyzed by SDS-PAGE as above.

### Reversed-phase high-performance liquid chromatography (RP-HPLC)

The concentrated eluate from on-column labeling of MI_N_C with I_C_-L (∼50 µg protein) was incubated with 1 µg Factor Xa (New England Biolabs) in oSB for 18 h at room temperature. Half of the reaction was analyzed by reverse phase HPLC using a Spirit Peptide C18 column (Aapptec: 5 µm, 25×0.4 cm) and Beckman System Gold equipment (Programmable Solvent Module 126, Programmable Detector Module 166, Software GoldV712). Mobile phase A was 0.1% TFA in water, while mobile phase B was 0.1% TFA in acetonitrile. A gradient was run (mobile phase B from 2% to 60% in 45 minutes), followed by 80% B for 5 min and re-equilibration with 2% B for 10 min. The flow rate was set to 0.8 mL/min and detection was done at both 210 nm and 497 nm. The peptide standard pep^Xa^ (sequence: GTLEGG; purchased from GenScript) at a quantity of 10 nmol was analyzed in a similar fashion.

### Mass spectrometry analysis (MS)

MS analysis was performed on (a) M^L^ protein excised from a Coomassie-stained SDS-polyacrylamide gel and digested with trypsin, (b) a standard *in vitro* labeling reaction of MI_N_C and I_C_-L without the liquid chromatography step, and (c) peptide products of a labeling reaction after digestion with Factor Xa (see above). For (a), the M^L^-containing polyacrylamide gel slice was reduced with DTT, carboxamidomethylated with iodoacetamide, and digested with trypsin (Promega, sequencing grade) for 7.5 h at 37°C. Peptides were extracted with 70% acetonitrile and 1% formic acid in HPLC-grade water. The reaction was automated on a ProGest digestion robot (Genomic Solutions). The extraction solvent was removed under vacuum using a speed vac, and tryptic peptides were resuspended in 30 µL of 5% methanol, 0.5% formic acid in HPLC-grade water. For (b), prior to sample injection, salts and excess I_C_-L peptide were removed from the labeling reaction sample using Zeba Micro Desalt Spin Columns (Pierce). For (c), ∼50 µg of a protein labeling reaction was incubated with 1 µg Factor Xa (New England Biolabs) in oSB for 18 h at room temperature.

LC-MS/MS was performed using an Ultimate pump and Famos auto-sampler (LC Packings, Amsterdam, Netherlands) interfaced to the nanoflow ESI source of a hybrid triple quadrupole linear ion trap (*Qtrap*) mass spectrometer (Applied Biosystems, Foster City, CA, USA). Samples (3 µL) were injected onto a capillary column (0.10×150 mm Chromolith C18, monolithic, Merck) at a flow rate of 1.2 µL/min. A linear gradient was run (5% solvent B to 35% B over 35 min), followed by 90% B for six minutes and re-equilibration at 5% B. Solvent A consisted of 2% acetonitrile, 0.1% formic acid in water, solvent B was 98% acetonitrile, 0.1% formic acid. The sample was sprayed through a distal coated fused silica emitter tip (75 µm ID with 15 µm ID tip, New Objectives *PicoTip*). The capillary voltage was 2.10 kV with a declustering potential of 60 V, the curtain gas was set to 15 (arbitrary units). Spectra were acquired using the Information Dependent Acquisition mode.

### Kinetic analysis of the labeling reaction

The MI_N_C precursor protein was incubated with the I_C_-L peptide in the presence or absence of TCEP at room temperature for 24 h, during which samples were removed from the reaction at specific time points, and the reaction was stopped by addition of SDS sample buffer. Samples were subjected to SDS-PAGE followed by Western blotting analysis using anti-C antibody (see above). Densitometry analysis of protein signals corresponding to MI_N_C and the I_N_C fragment was performed with the program ImageJ 1.342. Labeling efficiencies were calculated using the formula (AI_N_C/(AI_N_C + AMI_N_C))*100, where A_X_ represents the densitometry peak area of the respective protein signal. The amount of I_N_C fragment represented that of the spliced (labeled) protein, because these two protein products from the splicing reaction had essentially equal molar amounts (see Figure 4). Densitometry analysis for end-point efficiencies was performed similarly. For time-course analysis, the efficiencies were plotted as a function of time, and pseudo-first order rate constants (*k*_obs_) were determined as described for *trans-*splicing of the *Ssp* DnaE split-intein[36] and using the program KaleidaGraph 4.02.

### Activity of the fluorescence-labeled maltose binding protein (M^L^)

Amylose resin (New England Biolabs) was equilibrated with Amylose Column Buffer (ACB: 20 mM Tris-HCl, 200 mM NaCl; pH 7.4) and incubated for 1 h at 4°C with M^L^ protein. The resin was then washed extensively with ACB (sixty-times resin volume), and a small aliquot was viewed under a fluorescence microscope (AxioVert 200M, Zeiss) with excitation at 489 nm and a filter for emission at 520 nm. Proteins bound to the amylose resin were then eluted with ACB + 10 mM maltose, and samples were analyzed by SDS-PAGE, fluorescence scanning and Coomassie-staining as described above.

### C-terminal biotinylation of Enhanced Green Fluorescent Protein (E^B^)

Streptavidin-coated magnetic beads (Dynal Biotech) were washed three times with PBS-T (10 mM sodium phosphate, 0.9% NaCl, 0.1% Tween-20; pH 7.4) and incubated with either concentrated E^B^ protein (see above) or the soluble fraction from pTEI_N_C expression for 1 h at room temperature in the dark. Alternatively, beads were treated with or without 500 µM I_C_-B peptide for 1 h at room temperature before incubation with the soluble pTEI_N_C fraction in the presence of 0.1 mM TCEP for 18 h at room temperature. Unbound proteins were washed away, and beads were resuspended in PBS-T and spotted onto a glass slide for fluorescence microscopy as described.

### Labeling of transferrin receptor on live cells

CHO-TRVb cells were cultured in F12 nutrient mixture supplemented with 5% fetal bovine serum, 10 U/mL penicillin, and 0.1 mg/mL streptomycin at 37°C in a humidified chamber with an atmosphere of 5% CO_2_.

Actively growing cells (1.5×10^5^) were transfected with 0.5 µg plasmid DNA using the MicroPorator MP-100 (Digital Bio) with 10 µL Gold tips and the following settings: 1560 V, 5 ms pulse width, 10 pulses. Cells were seeded in 6-well plates and maintained as above in culture medium without antibiotics. For subsequent microscopy experiments, cells were seeded on sterilized cover slips.

For SDS-PAGE 24 h post-transfection, cells were washed once with PBS, scraped gently from the surface of the well into 1 mL PBS, and harvested by centrifugation (3,000×g, 2 min, RT). The cell pellet was resuspended in a lysis buffer (0.5% sodium deoxycholate, 1% Triton X-100, 1 mM EDTA, 150 mM NaCl, 50 mM Tris-HCl (pH 7.4) supplemented with a protease inhibitor cocktail (Bioshop) prior to use) and lysed by sonication (7 pulses at output 2).

For immunofuorescence, transfected cells were washed three times for 3 min each with 1 mL PBS, and fixed using 3.7% formaldehyde in PBS. After an additional wash step with PBS, cells were permeabilized with 0.05% Triton X-100 in PBS for 9 min at −20°C, and washed again with PBS. After blocking for 30 min with PBS-B (3% BSA in PBS), cells were incubated with primary antibody (α-HA diluted 1∶250, α-tubulin βIII both diluted 1∶1,000 in PBS-B) for 1 h at RT. After washing with PBS-B, cells were incubated with secondary antibody (goat anti-mouse IgG, conjugated with AlexaFluor 488 (Molecular Probes) and diluted 1∶2,000 in PBS-B) for 45 min at RT. Cells were finally washed three times with PBS, and coverslips were mounted on Superfrost Plus Microscopic Slides (Thermo Scientific) using Mowiol 4–88. Depending on the experiment, cells were counterstained for actin by adding rhodamine-phalloidin (10 ng/µL final concentration) to the secondary antibody solution. Nuclear DNA was sometimes stained by incubating coverslips with 100 nM TO-PRO-3 (Molecular Probes) for 5 min at RT prior to mounting. Cell imaging was performed on a LSM510 META Laser Scanning Microscope (Zeiss) at 100× magnification with excitation wavelength/band-path filter pairs (nm/nm) of 488/530 (Ar laser), 543/585 (HeNe laser) and 633/650 (HeNe laser), respectively.

Uptake of transferrin was assessed by incubating transfected cells 24 h post-transfection with tetramethylrhodamine-labeled human holo-transferrin (Molecular Probes), diluted to 15 µg/mL in F12 nutrient mixture containing 5% FBS, for 5–15 min at 37°C. Cells were then subjected to immunofluorescence as described above.

For receptor labeling, twenty-four hours post transfection, cells were washed twice with PBS, overlaid with labeling solution (buffer HBS (124 mM NaCl, 3 mM KCl, 5 mM D-glucose, 10 mM HEPES (pH 7.4), 2 mM CaCl_2_, 1 mM MgCl_2_) containing 0.1 mM TCEP and various concentrations of peptide I_C_-L) and incubated for 18 h at 23°C/5% CO_2_. Cells were then prepared for microscopy by fixation, permeabilization, and counterstaining with rhodamine-phalloidin as described above.
